# An adaptive prosthetic socket for people with transtibial amputation

**DOI:** 10.1038/s41598-024-61234-9

**Published:** 2024-05-15

**Authors:** Joan E. Sanders, Andrew C. Vamos, Joseph C. Mertens, Katheryn J. Allyn, Brian G. Larsen, Daniel Ballesteros, Horace Wang, Nicholas S. DeGrasse, Joseph L. Garbini, Brian J. Hafner, Janna L. Friedly

**Affiliations:** 1 Department of Bioengineering, University of Washington, 3720 15th Ave NE, Box 355061, Seattle, WA 98195 USA; 2 Department of Mechanical Engineering, University of Washington, 3900 E Stevens Way NE, Box 352600, Seattle, WA 98195 USA; 3 Department of Rehabilitation Medicine, University of Washington, 1959 NE Pacific St, Box 356490, Seattle, WA 98195 USA; 4 Department of Rehabilitation Medicine, University of Washington, 325 Ninth Ave, Box 359612, Seattle, WA 98104 USA

**Keywords:** Adaptive prosthesis, Auto-adjusting socket, Amputee comfort, Closed-loop control, Socket fit, Functional outcome, Translational research, Diagnostic markers

## Abstract

It is essential that people with limb amputation maintain proper prosthetic socket fit to prevent injury. Monitoring and adjusting socket fit, for example by removing the prosthesis to add prosthetic socks, is burdensome and can adversely affect users’ function and quality-of-life. This study presents results from take-home testing of a motor-driven adaptive socket that automatically adjusted socket size during walking. A socket fit metric was calculated from inductive sensor measurements of the distance between the elastomeric liner surrounding the residual limb and the socket’s inner surface. A proportional-integral controller was implemented to adjust socket size. When tested on 12 participants with transtibial amputation, the controller was active a mean of 68% of the walking time. In general, participants who walked more than 20 min/day demonstrated greater activity, less doff time, and fewer manual socket size adjustments for the adaptive socket compared with a locked non-adjustable socket and a motor-driven socket that participants adjusted with a smartphone application. Nine of 12 participants reported that they would use a motor-driven adjustable socket if it were available as it would limit their socket fit issues. The size and weight of the adaptive socket were considered the most important variables to improve.

## Introduction

Socket fit is reported as the single most important issue faced by people with lower limb amputation who wear prostheses^1,2^. Prosthesis users continually monitor their fit over the course of the day to determine if and when a fit adjustment is needed. For example, they may sense that their socket feels loose or unstable and determine that an adjustment is needed to make the socket fit better. They must decide the degree of adjustment by adding socks of different thickness or adjusting the panels of a manually-adjustable socket. Prosthesis users then assess the feel of the adjusted socket to determine if more adjustment is needed. Socket fit management is a never-ending process of monitoring and adjustment that many users find burdensome, drawing their attention away from other responsibilities and adversely affecting their quality-of-life.

Adaptive sockets are intended to sense changes in socket fit and make proper adjustments before the prosthesis user senses an issue. A number of strategies have been pursued^3–19^, the most common being the use of pressure sensing as a control variable and mechanical actuators to adjust socket size^3–15^. One of the first attempts to create an adaptive socket used a series of fluid-filled bladders and mechanical valves to adjust socket size^3^. Later, investigators pursued development of automatic size-adjusting sockets with powered actuators. Pressure sensors (and in one case shear sensors) were intended to be used for feedback control in most of these studies^4–15^. Pirouzi et al*.*^5^ tested their system on people with lower limb amputation, demonstrating that vertical limb displacement at the posterior brimline of the socket varied linearly with actuator pressure during standing cyclic weight bearing. These results suggested that actuator pressure could be used to control vertical limb displacement. A closed-loop control system designed for transhumeral prosthesis users was able to maintain consistent limb contact pressure on an able-bodied participant during lifting^9^, but no additional investigations were reported. Two novel manual systems, adjustable under open-loop control, were described^16,17^ and one shown in transtibial amputee participants to induce meaningful limb fluid volume changes during sitting.

Elevated vacuum, a technology that uses a small pump to maintain a vacuum between the limb and socket, is a form of automated pressure control^18,19^. It has exhibited mixed results in both clinical practice and scientific investigations. For example, elevated vacuum has been shown to maintain limb volume and enhance tissue health in some studies while in others it was found to increase risk of blister formation and be prone to pressure leaks^20–29^. Distance sensing, which monitors the distance between the prosthetic liner and socket wall as the residual limb changes size, has also been investigated as a control variable for socket size adjustment^30,31^.

A key challenge in designing the control system of an adaptive socket is to manage the conflicting design objectives—achieve stable coupling between the limb and socket while at the same time maintain health of the residual limb soft tissues. A tight socket may create good limb-socket coupling and facilitate stable ambulation, but if the residual limb atrophies or soft tissues are injured, the long-term impact can be detrimental to the user. An effective controller should enlarge the socket when the user’s residual limb increases in volume and decrease it when the limb decreases in volume. Research investigations using bioimpedance analysis to monitor limb fluid volume changes during prosthesis use showed that many participants, particularly those without co-morbidities, experienced limb fluid volume increases during ambulation^32–35^. Nearly all participants also experienced limb fluid volume increases when they released pressure on their residual limb during sitting^31,35–41^.

**Figure 1:**
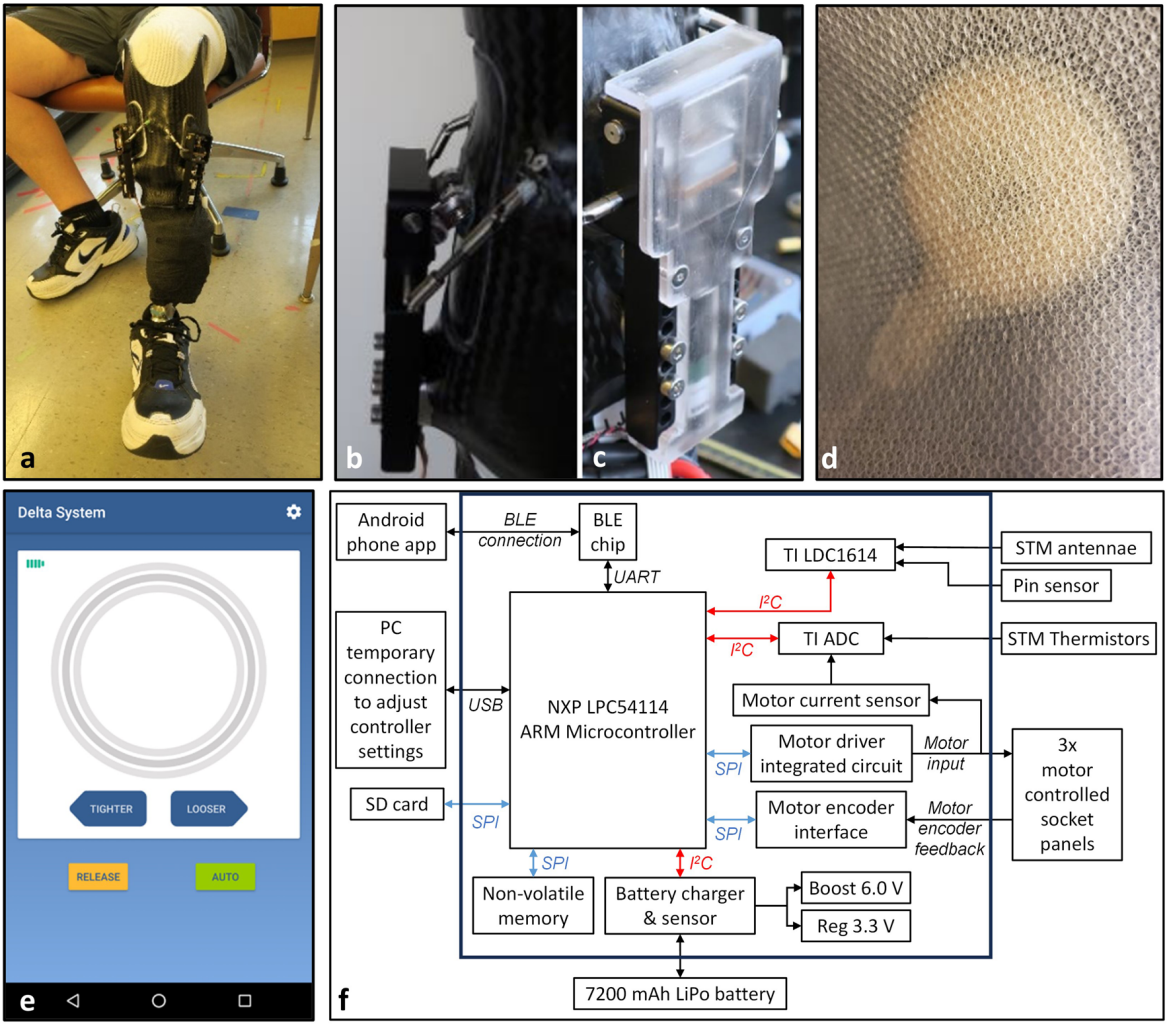
(**a**–**f**) Instrumentation. (**a**) Investigational prosthesis showing the anterior socket panels and adjustment mechanisms. The electronics box is underneath the black wrap on the pylon. (**b**) Side view of the adjustment mechanism showing the frame, actuator linkage, horizontal axis, and stabilization joints. (**c**) Close up of the mechanism, covered. (**d**) Antenna on the inside socket surface visible through the Nyglass. (**e**) Smartphone application showing buttons available to the user: TIGHTER, LOOSER, RELEASE, and AUTO. (**f**) Electronics schematic. The system operates the controller and collects and stores data from the sensors, motors, controller, and smartphone application. *ADC* analog-to-digital converter, *BLE* Bluetooth, *I²C* inter-integrated circuit, *SD* secure digital, *SPI* serial peripheral interface, *STM* socket distance sensor. All components are housed in a case fastened to the pylon and covered with vet wrap.

**Figure 2:**
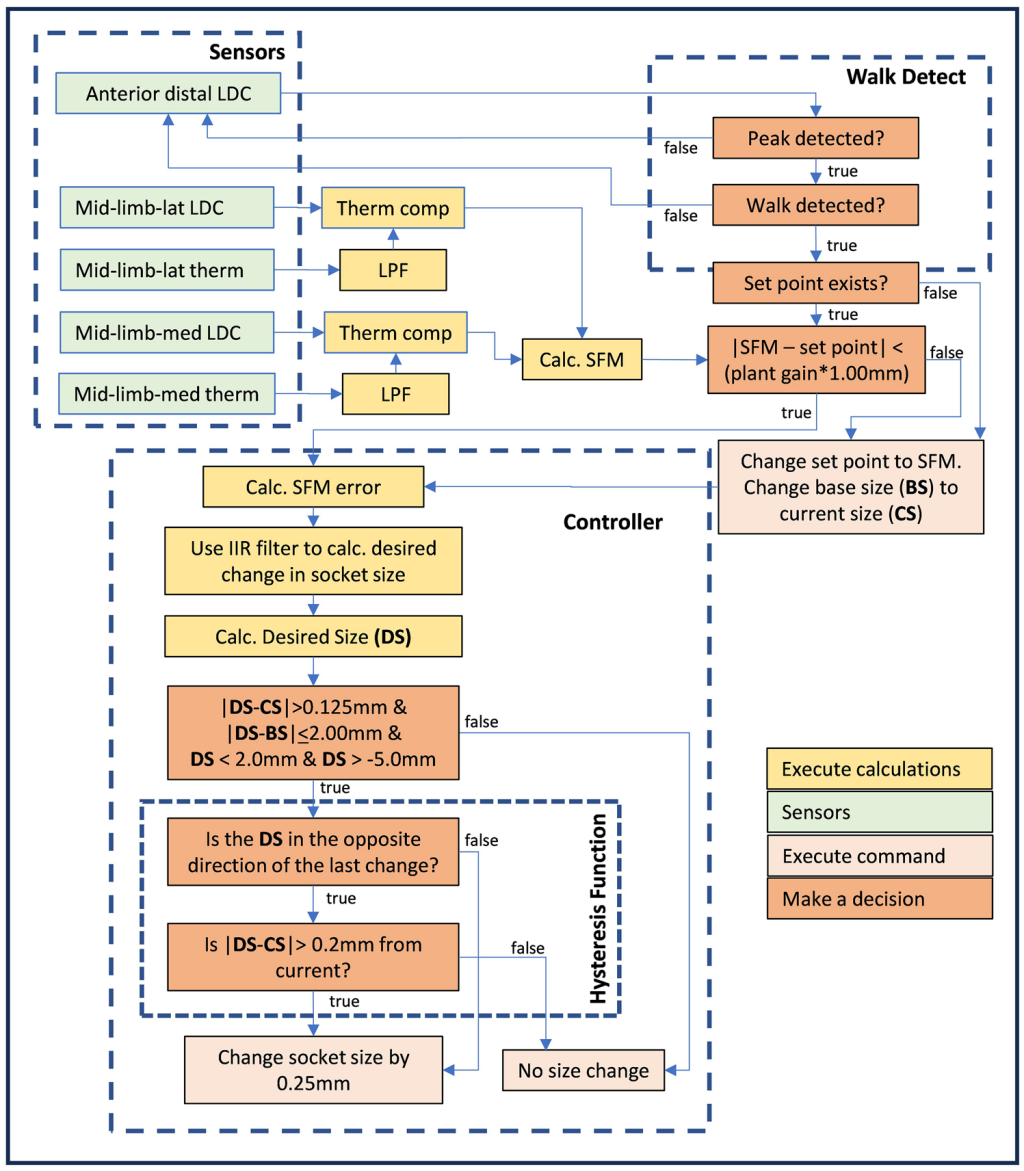
Flowchart for the controller. Data from the socket sensors is used to detect walking bouts and operate the controller that adjusts socket size. The controller is a proportional-integral controller and includes an IIR filter, limits, and a hysteresis function. Adjustment commands are sent to the motors that in this study all executed the same distance adjustment each time an adjustment was made.

In the present research an adaptive socket was tested that utilized these physiological responses of the limb to advantage. An investigational prosthesis with motor-driven adjustable socket panels was created to stabilize limb volume (Fig. 1a–c, Supplementary Video S1 online). Inductive sensor antennae embedded in the socket and a small amount of iron powder embedded in the elastomeric liner were used to measure limb motion in the socket and calculate a socket fit metric (SFM) (Fig. 1d). A microcontroller with interfaces to the socket sensors and to a smartphone application was positioned in a small enclosure fastened to the prosthesis pylon (Fig. 1f). A proportional-integral controller was implemented to adjust the radial positions of the panels based on the SFM (Fig. 2). The controller was active only during walking. Participants could use the smartphone application to release the socket panels during sitting and make small-step adjustments to socket size (Fig. 1e). The investigational prosthesis was also tested in a manual mode where only the smartphone application socket size adjustment was active. In locked mode, no adjustments were possible.

In this study, a randomized crossover trial was conducted with a group of participants with transtibial amputation to test the investigational prosthesis in auto, manual, and locked modes. We investigated how participants used the three different configurations under normal conditions and surveyed participants to assess their self-reported health outcomes. The performance of the controller to maintain the SFM was also characterized.

## Results

Twelve participants, 10 males and 2 females, with unilateral transtibial amputation took part in this study. Eleven participants had their amputation from trauma, and 1 for vascular insufficiency complications from diabetes mellitus. Eight residual limbs were conical in shape, 3 were cylindrical, and 1 was bulbous. Medians and ranges of participant characteristics and their activity metric data are summarized in Table 1, and individual characteristics are listed in Supplementary Table S2 online.

**Table 1:** Participant data. For the activity metric calculations, each participant's durations for a mode were calculated. The duration for a mode was defined as the participant's mean duration of the activity across all days in the mode.

Demographic characteristic	Median	Range
Age (y)	58	35–78
Time since amputation (y)	17	4–47
Body mass index (BMI*) (kg/m^2^)	26.7	21.2–39.2
Socket volume—mid-patellar-tendon to distal end (mL)	1332	805–1728
Residual limb length (cm)	15.9	9.0–19.0
Residual limb circumference (cm)	29.8	22.2–35.1

Activity metric	Low	High
Prosthesis day duration (h/day)	2.1	17.5
Time conducting walking bouts of 5 or more steps (h/day)	0.1	1.5
Doff time (h/day)	0.0	7.3
Step count (steps/day)	107	3360
Time wearing the prosthesis but not conducting walking bouts of > 5 steps (% of prosthesis day)	34.8	98.8

*Amputee Coalition. About body mass index (BMI), 2016, Washington, DC: Amputee Coalition. https://www.amputee-coalition.org/limb-loss-resource-center/resources-filtered/resources-by-topic/healthy-living/about-bmi/. Amputee Coalition, Washington, DC. Accessed April 7, 2022.

### Activity

**Figure 3:**
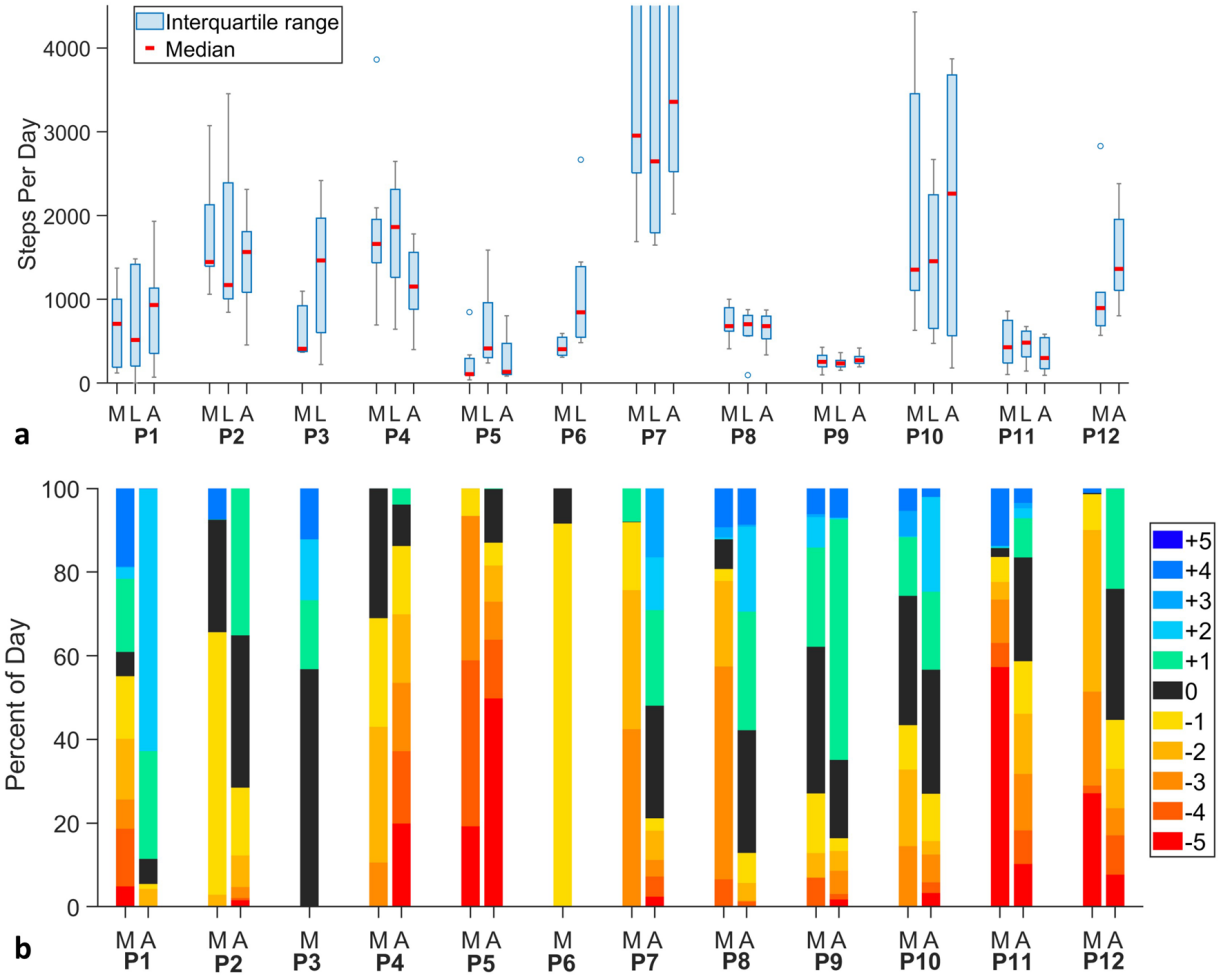
(**a**,**b**) Prosthesis use results. (**a**) Step count per day for each mode. The blue boxes indicate the interquartile range, the gray bars the maximum and minimum, and the blue circles the outliers. The red lines are the median steps per day. The figure is zoomed in to highlight differences between modes for each participant, but this did cut off the upper range for participant 7. (**b**) Percentage of total prosthesis day time at each panel position. Medians for the manual mode (M) and the auto mode (A) are shown for each participant. Participants tended to have a larger socket size (higher panel positions) for the auto mode compared with the manual mode.

For all participants, the highest step counts were achieved in either auto mode or locked mode, not manual mode (Fig. 3a). In general, high-activity participants were most active in auto mode, and low-activity participants were most active in locked mode. Of the ten participants who tested auto mode, five of the six most active (1,2,7,10,12) showed higher step counts in auto mode compared to the other modes. Three of the four least active participants who tested auto mode (5,8,11) showed higher step counts in locked mode than the other modes. Both participants who tested only manual and locked modes (3,6) showed higher step counts in locked mode.

**Figure 4:**
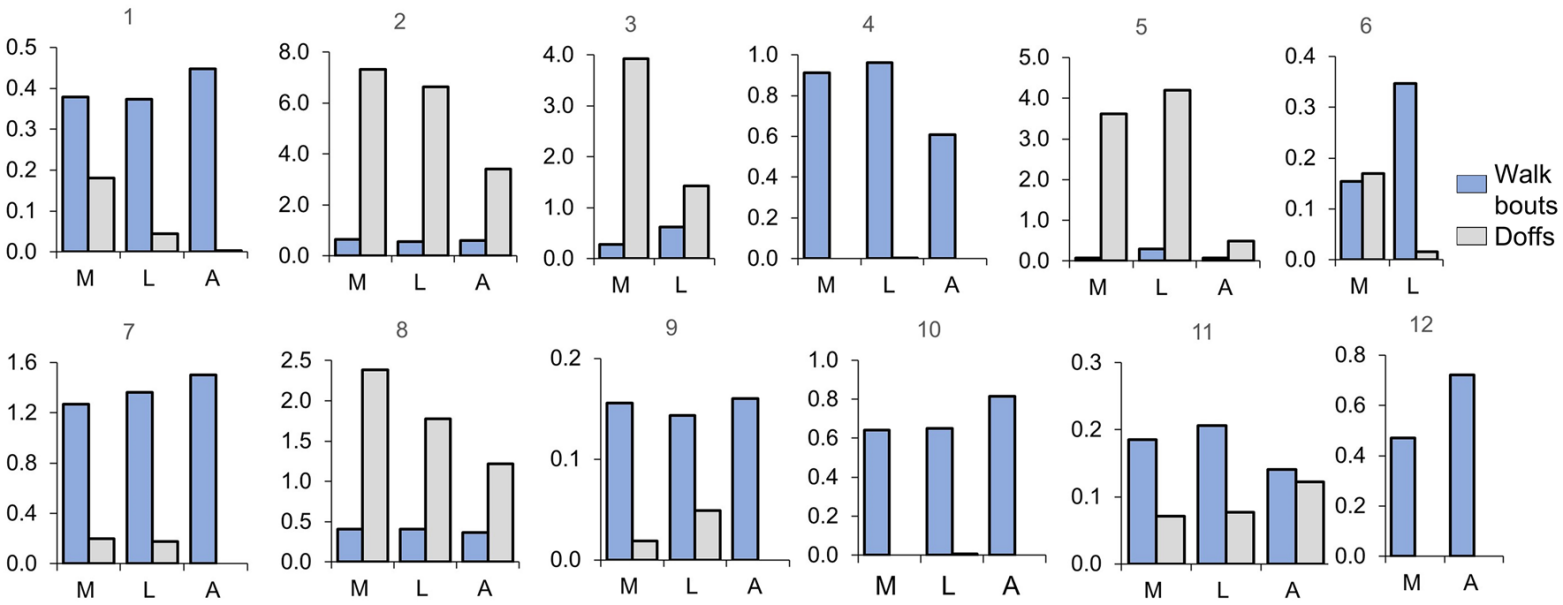
Median daily walk bout times and doff times for all participants (*n* = 12). The *y*-axis is median time/day in hours. The blue bars show walking bouts, and the gray bars show doffs (Stands, sits, and weight shifts are not shown). The three modes are shown on the *x*-axis: M: manual; L: locked; and A: auto.

Some of the other activity metrics followed the trends seen in step counts. Of the six participants who increased their daily step count in auto mode compared with the other modes (1,2,7,9,10,12), five of those participants (1,7,9,10,12) also increased their daily walk bout time in auto mode compared to the other modes (Fig. 4). Four of the six participants (1,2,7,9) decreased their median daily doff time in auto mode. Both participants who tested only locked and manual modes had higher median daily walk bout time and less time doffed in locked mode than manual mode.

Prosthesis day duration did not follow the trends seen in step counts. Three (1,2,10) of the six participants showed a shorter median prosthesis day in auto mode, and three (7,9,12) showed a longer median prosthesis day in auto mode (Table 2). For the two participants who tested only locked and manual modes, participant 3’s median prosthesis day was comparable for the two modes while participant 6’s was lower for manual mode than locked mode.

**Table 2:** Median prosthesis day duration by mode (h).

Partic	Manual	Locked	Auto
1	4.7	7.2	5.9
2	13.5	11.6	9.5
3	15.8	15.6	–*
4	14.7	12.3	12.2
5	12.7	14.3	2.3
6	7.9	13.3	–**
7	14.5	15.3	16.8
8	15.1	14.8	15.1
9	16.8	16.4	17.5
10	11.1	12.5	9.5
11	2.1	7.5	2.8
12	11.0	–***	14.7

*Participant 3 could tolerate only minimal inward panel motion from flush, plant gain not measurable.

**Participant 6 did not use the auto mode because of clinical concerns about cognitive issues.

***Participant 12 did not execute locked mode because of potential suspension issues from his high sock ply.

### How participants used the prosthesis

Four participants changed how they used their prosthesis in the different modes, illustrating other impacts of mode on user performance:

- One participant (5) meaningfully increased his percentage of bouts that were longer than 20 s in auto mode compared with the other modes (Supplementary Fig. S3 online). However, he also showed a shorter median prosthesis day, lower daily walk bout time, and lower daily step count in auto mode (Table 2, Figs. 3a, 4), possibly because he tired from the longer bouts.
- One participant (8) demonstrated relatively consistent prosthesis day duration, daily walk bout time, daily step count, and bout duration medians across modes (Table 2, Figs. 3a, 4, Supplementary Fig. S3 online). However, her median doff time was lower for the auto mode compared to the other modes, and her residual limb was deeper in the socket by 1 to 2 pin notches (3 mm/notch) for the auto mode. These results suggest that panel adjustment in auto mode improved fit such that she felt more comfortable deeper in the socket.
- One participant who walked more than 20 min/day for each mode (4) demonstrated a lower daily walk bout time and daily step count in auto mode compared with the other modes (Figs. 3a, 4). As evident by her relatively high number of button presses in auto mode and her frequent 1-h disabling of the controller (Supplementary Fig. S3 online), this participant “fought” the controller. She was the only participant who demonstrated this behavior and she was the only participant with a bulbous limb, suggesting a different controller is needed for participants with this limb characteristic.
- One participant (11) executed many more button presses in auto and manual modes than any other participant (Supplementary Fig. S3 online), though his median daily time conducting walking bouts of 5 or more steps was < 13 min/day in all three modes (Fig. 4). These results suggest that the participant valued adjusting the socket size during sitting possibly to improve comfort.

### Button presses

In general, participants pressed the buttons on the smartphone application to adjust socket size less when the socket was in auto mode than when it was in manual mode (Supplementary Fig. S3 online). Participant 4, who “fought” the controller, was an exception. Part of the reason the number of button presses was so high for most of the participants is we did not put an auto-return feature in the application. This was done intentionally to focus user feedback instead on presence and non-presence of auto adjustment while walking. Participants executed individual step adjustments (button presses) to return the panels near to their prior position after a socket release. We also noted that, in general, participants’ socket sizes were larger for the auto mode than the manual mode (Fig. 3b). This result suggests that in manual mode participants over-reduced socket size beyond that necessary to maintain a consistent sensed distance.

### Controller operation

**Figure 5:**
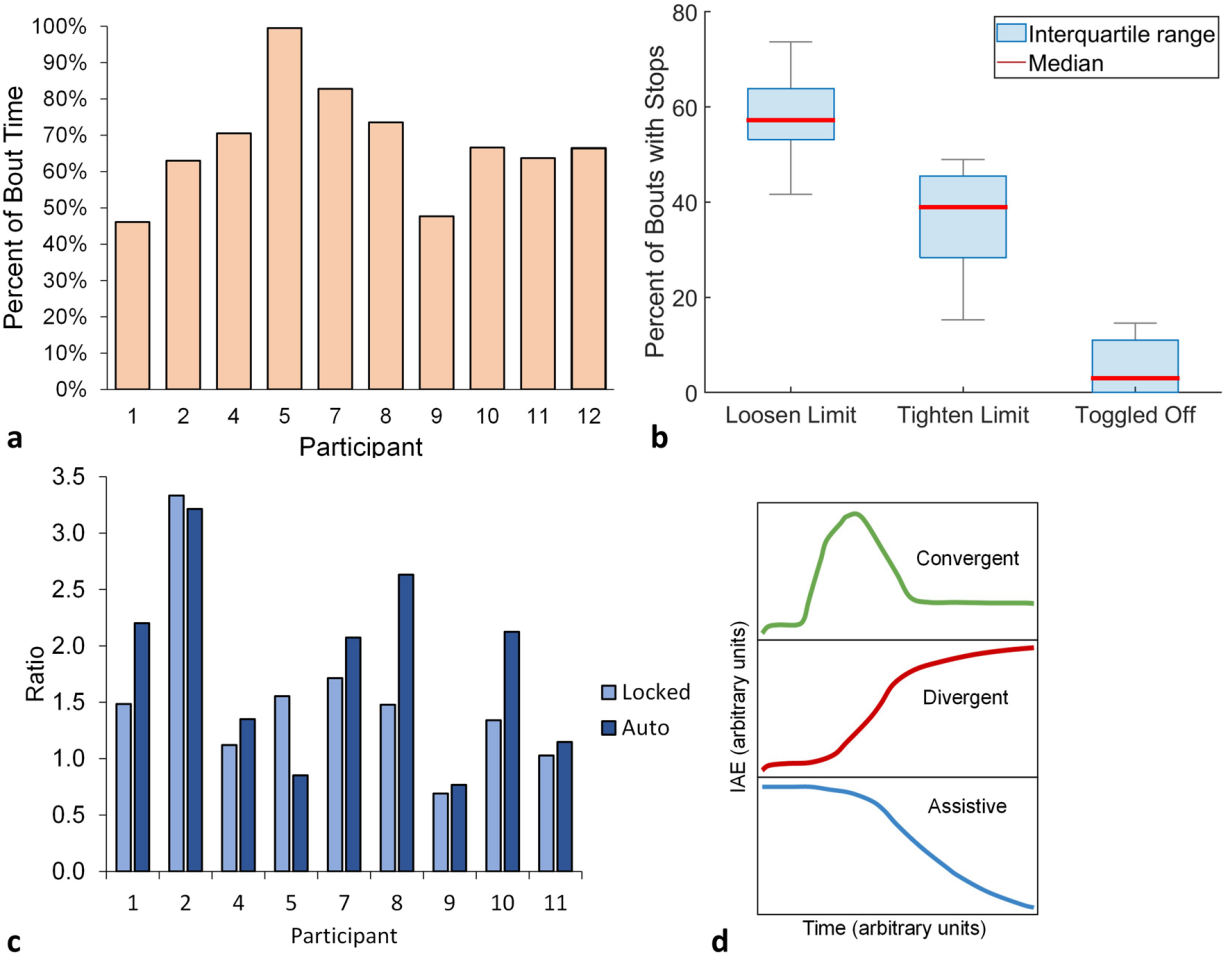
(**a**–**d**) Controller performance. (**a**) Percentage time that the controller was active, i.e., not stopped by the controller rules nor the participant. (**b**) Percentage of bouts for which panel adjustment was temporarily stopped. Loosen limit: Adjustments stopped because controller rules were violated at the loosen limit. Tighten limit: Adjustments stopped because controller rules were violated at the tighten limit. Toggled off: The participant turned off the controller with the smartphone application. The gray bars indicate the maximum and minimum. (**c**) Quotient of the sum of the number of convergent and assistive bouts divided by the number of divergent bouts. (**d**) The three classifications of socket fit metric (SFM) response curves when the controller was activated included: A convergent response (green) was a rise to a maximum followed by a decrease that over time approached an approximately constant value, where the constant value was of greater magnitude than at the beginning of the bout. A divergent response (red) was a rise over time that did not maximize. An assistive response (blue) was a decrease over time that may or may not have reached a constant value.

In auto mode, the system was under active control a mean of 68% (range 46–100%) of the time participants were walking (Fig. 5a). Active control means that the controller was free to make adjustments and was not stopped by rules programmed into the firmware to avoid a rapid large adjustment or by participant intervention. For cases where panel adjustment was temporarily stopped, the most frequent reason was because the controller rules at the panel loosen limit were violated (median 57.2% of the total time, range 41.7–73.7%), followed by because the controller rules at the panel tighten limit were violated (median 38.9%, range 15.3–48.9%) (Fig. 5b). Panel adjustment was also stopped, albeit rarely, because participants temporarily turned off the controller (median 3.0%, range 0.0–14.6%).

The operational effectiveness of the controller was judged by its ability to maintain the SFM at the set point. Convergent and assistive responses, as defined in Fig. 5d, indicated more favorable performance than divergent responses. The ratio of the number of bouts with convergent and assistive responses to the number of bouts with divergent responses was a median of 2.1 (range 0.8–3.2) for auto mode and 1.5 (range 0.7–3.3) for locked mode (Fig. 5c). All participants demonstrated higher ratios for the auto mode than the locked mode except for participant 5 who showed a 1.8 × higher ratio for locked mode than auto mode, and participant 2 who showed a comparable ratio for locked and auto modes.

### Self-reported outcomes

**Figure 6:**
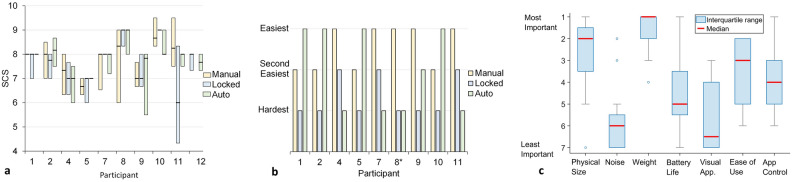
(**a**–**c**) Participant self-report data. (**a**) Socket comfort scores (SCS) for manual, locked, and auto modes. (**b**) End of study rankings of the easiest, second easiest, and most difficult mode to maintain socket fit. *: Participant 8 ranked the auto and locked mode both as most difficult. (**c**) Participant end-of-study rankings of investigational prosthesis features. ‘1’ is most important and ‘7’ is least important. The blue boxes indicate the interquartile range, the gray bars the maximum and minimum, and the blue circles are outliers. The red lines are the medians. Visual app. = visual appearance.

Median socket comfort score^42^ (SCS), which is a self-report rating from 0 to 10 of how the socket feels at that moment, collected three times per day during take-home testing, showed differences between modes of less than or equal to 1 for all participants except participant 11 whose median locked mode rating was approximately 2 points lower than that of the other modes (Fig. 6a). All 9 participants who used all three modes reported that either the auto mode or the manual mode was the best at maintaining fit (Fig. 6b). Six of the 9 participants rated the locked mode the worst at maintaining fit.

Most participants’ (6 of 9) median SCS scores collected during take-home matched their responses to questions asked at the end of the study, demonstrating consistency. They ranked either the auto mode (1,2,5) or the manual mode (4,7,11) best at maintaining socket fit. However, three participants demonstrated inconsistency (8,9,10)—median SCS scores did not match end-of-study responses.

Participants rated the physical size and the weight of the adaptive prosthesis as the variables they considered most important (Fig. 6c). Noise and visual appearance were considered the least important. The relative importance of battery life, ease of use, and smartphone application control varied across participants.

Nine of the 12 participants agreed or strongly agreed that they would use a prosthesis that allowed smartphone application to make socket size adjustments; the capability to make socket size adjustments using the application would limit their socket fit issues; and they would like the application to monitor socket fit (Supplementary Table S4 online). While only 7 participants agreed or strongly agreed that they would like to be provided the socket fit data, 10 participants agreed or strongly agreed that they would like to share the socket fit information with others, primarily their prosthetist. Participants were divided on whether they would prefer a remote-control fob over the smartphone application, 3 agreed or strongly agreed, 6 neither agreed nor disagreed, and 3 disagreed or strongly disagreed.

Six themes were identified from the open-ended interview questions, and the results are summarized in Supplementary Table S4 online. Participants voiced challenges introduced by the physical size of the socket, e.g., running into things and difficulty wearing pants over the adaptive socket. They wanted an easy means of seeing the current battery power, for example a battery bar icon in the smartphone application. Participants suggested that the application should have a set position button, i.e., the panels would move to that user-programmed position upon a single button press. One participant said that he would prefer auto-release during sitting rather than having to manually release the panels. Two participants voiced that the controller was too sensitive, feeling that it adjusted socket size too often. The participants preferred an easy-to-access means for adjustment other than the smartphone application, for example buttons on the socket or a fob that clipped onto the socket. Participants were positive on the controller and/or the capability to adjust the socket themselves. One participant voiced a preference for adding a flexible inner liner.

## Discussion

Sockets that use mechanical ratchets, levers, hand pumps, or other mechanisms to adjust socket size are becoming commonplace in lower-limb prosthetics. They are an advancement over the traditional strategy of changing sock thickness to manage limb volume fluctuation and other socket fit issues. The purpose of this research was to extend size-adjustable prosthetic socket technology into two new directions—motor-driven adjustment with a smartphone application and automated adjustment to maintain socket fit. These advances have the potential to enhance prosthesis users’ quality-of-life by reducing the mental and physical burdens surrounding routine socket fit adjustments.

Motor-driven panels help reduce the time of the adjustment process to seconds instead of minutes because the user does not need to stop moving to make adjustments. The cyclic process of stopping, changing the socket size, and walk-testing the new fit disrupts prosthesis users’ lives, particularly when the process must be done repeatedly to achieve a proper fit. All participants in the present study were able to make socket size adjustments (manual mode) or have adjustments made for them (auto mode) while walking. As expected, when asked which mode made maintaing socket fit easiest, all participants preferred an adjustable mode over the locked mode (Fig. 6b). The mechanical design of our adjustable socket may also have facilitated participants’ abilities to maintain a stable gait during adjustment. The mechanism used a relatively small step size (0.25 mm), an approximately 0.22% socket volume change for each smartphone application button press. For reference, an experienced prosthetist consistently detected socket volume errors of 1.0% in a study testing the accuracy of computer-manufactured sockets^43^.

Unlike prior adaptive socket strategies^3–15^, our control system was designed to maintain a consistent sensed distance, not pressure. We used sensed distance because it showed the capability to pick up socket fit changes before participants or practitioners^44^, and changes in sensed distance followed changes in limb fluid volume^45^. Pressure sensing may have a role in socket design from a long-term monitoring perspective^46,47^. However, before pressure sensing is used for real-time feedback control in adaptive sockets, its capability as a quick-response, highly sensitive metric of socket fit needs to be demonstrated. Pistoning on the bottom of the limb is also a candidate for real-time feedback control^48^.

Results from this study suggest that participants changed their activity and/or how they used the investigational prosthesis when wearing an adjustable mode (manual or auto) compared with the locked mode. The six participants who increased their daily step count in auto mode compared with the other modes (1,2,7,9,10,12) (Fig. 3a) may have done so because of an improved socket fit and/or they had more energy. Long-term testing would be needed to determine whether these participants maintained this pattern of activity.

It is possible that some of the prosthesis use changes observed in the present study resulted from the novelty of the technology and the relatively short duration of take-home use. Issues that may arise during routine clinical use are unknown. The results are nevertheless encouraging and as discussed below provide insight towards the design of larger clinical studies and enhancement of adaptive prosthetic sockets.

Participants’ change in doff behavior for the adjustable (auto or manual) vs. non-adjustable (locked) modes (Fig. 4) points to the potential relevance of quick socket-release technology in clinical care. Auto mode reduced doff time perhaps because it created a better socket fit but also because the participants could execute socket release without doffing. Doffing, one form of socket release, has been shown to facilitate fluid volume stabilization for prosthesis users who otherwise tended to lose fluid volume over the day^35–37^. Socket release without doffing, releasing socket panels with or without pulling the residual limb a few cm out of the socket, has also been shown to facilitate limb fluid volume stabilization^31,39,40^ and is much easier to execute than full doffing. If prosthesis users were to integrate temporary socket panel release into their everyday routine or it were to be executed automatically by the controller, they may experience less limb volume fluctuation and need to doff their prosthesis less often.

The data from this study indicated that manual mode did not facilitate greater walking activity than either auto or locked modes for any participant. This may have been in part because of when participants chose to make adjustments and how much adjustment they chose to make. An advantage of the auto mode is that the controller monitoring the SFM detected changes in socket fit well before participants did^44^, and the controller made small, automated adjustments based on the SFM data to maintain the socket fit. In manual mode, participants made adjustments only when they sensed a detrimental change in fit. In preliminary testing leading up to the present study, we found that some participants did not detect changes in fit until 20 to 30 min after a change in SFM occurred. Further, participants using manual model may not have made appropriate size adjustments after sensing a socket fit issue, which could have exacerbated their socket fit problem. A rigorous clinical investigation is needed to further explore if smartphone-controlled motor-driven adjustable socket technology (manual mode) improves health outcomes in only some but not all users, and if so, what patient characteristics need to be considered when making the decision to use this type of technology. The simple and quick adjustment capabilities of manual mode may have both positive and detrimental effects, and this possibility needs investigation. It is likely that user education and training will affect outcomes and will need to be carefully planned.

Though we did not include a return-to-the-prior-socket-size button in the smartphone application in this study, we believe this feature would likely be welcomed and used by participants. Practitioner concerns about implementing socket release technology on patients whose residual limbs may excessively swell could be alleviated by designing size limits into the firmware, allowing practitioners to limit panel radial outward adjustment. The interpretation that socket release technology is likely to be successful in regular clinical use is conjecture, and additional clinical testing would be needed to confirm or refute this hypothesis.

The larger socket size in auto mode (Fig. 3b) is consistent with the intended operation of the controller to enlarge the socket when the posterior mid-limb moved closer to the socket wall, a limb fluid volume increase, an opportunity to stabilize limb fluid volume. In manual and locked modes, participants may not have sensed this change and did not make size adjustments. There was no evidence that participants felt less stable in auto compared to the other modes, nor that they experienced any type of skin injury. An interesting question that needs exploration is to what degree does a larger socket size, both during walking and resting, improve participants’ activity outcomes.

The controller turned off about 32% of the time it was in active mode. The temporary shut offs were caused primarily by an excessively rapid or large size change instruction sent to the controller, triggering a rule violation. We have found since completing this study that most prosthesis users tend to experience a decrease in their SFM over approximately the first 15 steps after donning their socket, whether those steps are executed in a single bout or distributed over several bouts. We suspect the SFM decrease is from the residual limb sinking deeper into the socket—most participants reached a more stable depth in about 15 steps. SFM decreases shortly after donning may help explain why some of the temporary shut offs were from rules violated at the loosen limit as opposed to the tighten limit (Fig. 5b).

The result that the ratio of the number convergent and assistive bouts to the number of divergent bouts was in general higher for auto mode than locked mode (Fig. 5c) suggests that the controller performed as intended. It is unknown why the ratio was so much lower for participant 5, though we do note that his plant gain was much higher than other participants (Supplementary Table S2 online). Possibly, unique physiologic qualities reflected in the participant’s plant gain necessitated use of a different control strategy for this individual. Investigation of the reasons may guide enhancements to future control systems that better meet individual user needs.

If the set point was far from the current SFM value before automatic control was initiated at the start of a walking bout, the controller set point was set equal to the current SFM. This action, necessary to avoid sudden large changes in the controlled socket size, caused the set point to change over the day. It is currently unknown if a fixed set point would have been more favorable. We believe that analysis of the data between walking bouts may provide insight into how to adjust the set point after a reset. For example, if participants primarily stood between walking bouts they may then have the potential for limb fluid volume increase once they start walking again^34^. Thus, a slow set point increase after the bout starts may be appropriate. The challenge is to identify strategies that adjust the set point properly so as to enhance participant satisfaction and performance, given the knowledge that some participants prefer tight sockets during activity and others prefer loose sockets. We did not design the controller to make socket size adjustments during standing because of the relatively low vascular drive during standing and thus minimal capability for limb fluid volume gain. A system that continually decreased socket size during standing would likely be unfavorable to limb volume stability and health.

Participant preference for one adjustable mode over the other (auto over manual, or manual over auto) may have been, in part, a psychological issue. This interpretation may help explain inconsistencies in SCS scores and interview data collected at the end of the study. In studies leading up to the take-home testing conducted here, some participants anecdotally suggested they were nervous with an automated controller making adjustments, preferring instead to control socket fit themselves. Some believed with confidence that they would do better maintaining fit in manual mode than the controller in auto mode. Others had no concern and welcomed passing the responsibility of socket adjustment off to the controller. It would be expected that in clinical practice, this issue will need to be considered by prosthetists when prescribing adaptive sockets. One participant (7) who demonstrated more favorable activity results for the auto mode than the other modes did not voice preference for auto mode, in part because he believed at one point while in auto mode he experienced the controller reducing socket size while driving. Though inspection of the data after the study showed no such control system behavior, the experience caused him some anxiety about auto mode and caused him to indicate preference for the manual mode.

Participants were often more interested in having their socket fit data sent to others, e.g., their prosthetist, than seeing and using the data themselves. This result is consistent with the desire to minimize distraction wearing a prosthesis. Presumably, participants saw value in the socket fit data and wanted their practitioner to use the data in their clinical care.

The primary limitations in this study were the protocol challenges introduced by IRB restrictions because of the COVID-19 pandemic. We would have preferred participants use the investigational socket for a month in each mode, for ordering to be randomized, and for washout periods to be included. However, these were not possible due to IRB restrictions.

The size and weight of the adaptive socket, deemed by participants to be the most important qualities to improve, could be reduced by removing the side U-joints, reducing the size of the frame and mechanism, and improving the power efficiency of the motors. However, given that this is the first reported take-home test of a controlled size-adjusting and manual smartphone-adjustable socket, the findings are useful to set next steps in development and create test strategies for long-term studies.

Having the flexibility to operate off both a smartphone application and a fob capable of mounting to the prosthesis would likely enhance acceptance and satisfaction of adaptive sockets by prosthesis users. Auto socket release upon sitting is an appropriate next step in development and testing the clinical performance of adaptive sockets in longer studies and will further enhance the capability of this technology to enhance the lives of people with limb amputation.

The key concepts learned in this study should be applicable to other adaptive sockets and levels of amputation. Future research should carefully consider what participant population each design is intended to serve and create product interfaces, training strategies, and education materials that match users’ individual needs.

## Methods

### Background

In prior work leading to this study, we used a custom limb fluid volume monitoring system that implemented a bioimpedance analysis modality^49^ to investigate how residual limb fluid volume responded to changes in socket size. Data collected using this tool and studies leading up to them helped provide the physiologic understanding needed to develop an adaptive socket^17,29,31–40,45,50–52^. In addition to demonstrating to us that socket size increases during walking and sitting could facilitate residual limb fluid volume stabilization, particularly in participants without co-morbidities, these studies indicated that the distal area of the residual limb needed to be unloaded for release-relock during sitting to be effective. This information helped establish the appropriate number of panels and their dimensions. Within a test session, participants' range of acceptable socket size (maximum percent socket volume change relative to neutral—minimum percent socket volume change relative to neutral) was on average about 5.5% (range 2.5–7.6%). This information helped establish the appropriate radial distance to move the panels. Most participants experienced greater fluctuation in their rate of limb fluid volume change early compared to later in the day, indicating that greater socket size adjustment should be expected in the morning than in the afternoon. A socket of consistent volume but variable shape may affect limb fluid volume, making it difficult to use such a socket in closed-loop control. This information encouraged us to design a socket that changed volume without meaningfully changing shape. The practice of locking the pin before tightening the panels may allow greater space for the limb in the distal socket and thus better retain limb fluid volume than when tightening the panels first and then locking the socket. This information helped establish an important operational capability in manual mode.

Also prior to this study, we used a cabled three-panel adjustable socket with a single motor mounted underneath the socket and adjusted panel position while participants walked wearing the investigational prosthesis^53^. Our results showed an approximately linear relationship between socket size (panel radial distance) and limb fluid volume as well as between socket size and the distance sensed between the liner and socket at posterior mid-limb locations^30,45^. Relationships to sensed distance at other locations or to pin sensor data were not as strong. We selected the mean distance measured from two posterior mid-limb sensors during stance phase as our SFM and implemented it in a proportional-integral controller^30^. The error between the SFM and the set point averaged 0.003 mm^30^, corresponding to an approximately 0.08% socket volume error, considered clinically acceptable.

Also prior to this study, we replaced our original cabled-panel design with one actuator per panel^31^. This design was lighter and overcame issues with the three panels floating, i.e., moving altogether under a single cable suspension (constant cable length), which could lead to inconsistent panel movement. Further, by moving the horizontal rods on the back sides of the panels radially inward and outward (with respect to the residual limb) to accomplish adjustment and driving each panel with its own motor, we ensured that at a single setting, the socket volume and shape were relatively consistent. This design overcame limitations of inconsistent socket volume and shape as well as eliminated the risk of stress concentrations delivered to the residual limb at the top and bottom of each panel.

Using this system in a prior study, the controller maintained stable operation during 98% of the walking bouts tested^31^. We also found in the same study that releasing the socket panels during sitting facilitated limb fluid volume stabilization, a result also shown for partial and full doffing^31,35–37,39,40^. The findings from this prior work were implemented in the design of the present controller. In the present study, the length of the actuator frame was reduced to 11.2 cm and the maximum width to 4.8 cm. Weight of each actuator was reduced to 116 g.

### Participant inclusion and exclusion criteria

Participants 18 years or older were included if they had a transtibial amputation at least 18 months prior, were using a properly fitting socket for at least 6 h a day and were a Medicare Functional Classification Level^54^ K-3 ambulator (community ambulator who has the ability to traverse most environmental barriers and may have vocational, therapeutic, or exercise activity that demands prosthetic utilization beyond simple locomotion) or higher. Needing to use a walking aide (e.g., a cane) or presenting with active skin breakdown excluded participants from the study. A University of Washington Institutional Review Board approved all study procedures (Study #49624), and written informed consent was obtained from participants before study procedures were initiated. All methods were performed in accordance with the Declaration of Helsinki—Ethical Principles for Medical Research Involving Human Subjects.

### Investigational prostheses

An adaptive socket with motor-driven adjustable socket panels was fabricated for each study participant. The prosthesis was completed using pin lock suspension and the foot from the participant’s traditional prosthesis or a standardized energy storage and return foot (LoPro 81, RUSH, Proteor, Tempe, Arizona) if the foot could not be removed or fit in the investigational prosthesis.

The investigational sockets were duplicate in shape to participants’ traditional sockets but included three adjustable panels. The traditional sockets were scanned using a high-resolution coordinate measurement instrument with a thin long stylus arm (FaroArm Platinum, FARO Technologies, Lake Mary, Florida) and a surface was made from the point cloud data using computer-aided design software packages (Geomagic, Design X, 3D Systems, Research Triangle Park, North Carolina; OMEGA Tracer, WillowWood, Mt. Sterling, Ohio)^48^. Foam positives of the scanned shapes were fabricated using a carver (C7, Provel, Cle Elum, Washington), and carbon-fiber layups were fabricated over the positives.

Panels were cut out of the sockets at the anterior medial flare, anterior lateral flare, and posterior midline. Panel sizes were maximized so as to impact socket volume change while avoiding bony prominences such as the anterior distal tibia, fibular head, and tibial crest that are known to be sensitive to compression. Each panel was mounted to a motor-driven leadscrew and actuator linkage positioned within a frame fastened to the socket (Fig. 1a–c). The motors (1724A006SRIEH2-4096 + 15A 152:1 + MG03) drove the leadscrews and actuator linkages, causing the panels to translate in the radial direction. A step size for adjustment of 0.25 mm radial movement was selected because it was sufficiently high to produce a meaningful change in socket volume but not so high as to induce an unstable gait. The root-mean-square error of the motor position was < 0.0015 mm. The displacement range of the panels was 5.0 mm inward and 4.5 mm outward relative to flush. This range corresponded to a median socket volume change from -4.4% to + 3.9% for the participants in this study. Each panel was attached to the actuator linkage via a rod that allowed the panel to rotate about a horizontal axis parallel with the socket tangent, ensuring that the edges of the panel at the top and bottom did not protrude into the residual limb. No flexible inner liner or pads on the panels were used. The outside dimensions of each motor and frame assembly were 11.2 cm length, 4.8 cm width, and 1.7 cm thickness. Including the anchors embedded in the socket and the stabilization brackets on the sides of the panel, each motor/actuator linkage/frame assembly weighed 160 g.

Inductive sensor antennae were embedded within the socket wall during fabrication (Fig. 1d) to sense the distance between the socket and a magnetically-permeable target in the liner (iron powder) (Supplementary Fig. S5 online). A capacitor was connected across each antenna to form an inductor-capacitor (L-C) unit. A thermistor placed local to the antenna was used to record temperature for thermal compensation. When the L-C unit was powered by an inductive sensing chip (LDC1614, Texas Instruments, Dallas, Texas), the presence of the magnetically permeable iron local to the antenna reinforced the inductor and lowered the sensor’s oscillation frequency in a distance-dependent manner. The change in frequency measured by the inductive sensing chip was a sensitive measurement of the distance between the sensor and target.

Two sensor antennae were positioned posterior mid-limb, one medial and one lateral, and a third sensor was positioned anterior distal. The sensed distance data were used to operate the controller. Elastomeric liners with a trace amount of iron powder in the elastomer next to the fabric backing were produced, either by a manufacturer that we contracted to make them (WillowWood, Mt. Sterling, Ohio) or by our team replacing the fabric backing of a commercial liner with a custom fabric backing that included the iron powder^55^. The sensors were calibrated in a two-step process. A bench calibration procedure was executed by displacing an antenna from the target in the liner in discrete steps using a high-resolution vertical height gauge. Balloon calibration, where a silicone model of a residual limb was placed inside the liner and that assembly was placed within the socket and pressurized to 35 kPa (locking pin engaged), was used to determine the *y*-offset at each sensor location (the radial distance of the antenna from the surface). Thermal calibration was conducted with the sensors in an oven heated across a 38 °C range reflecting temperatures encountered during clinical use.

Since the vertical position of the limb in the socket was expected to be meaningful to socket fit, an additional sensor was used to record the depth of the locking pin in the shuttle lock at the bottom of the socket. The sensing element was a wire coil wrapped around a former and embedded in epoxy^56^. The locking pin extending from the bottom of the elastomeric liner passed through the center of the coil. An inductive sensing strategy was implemented. The signal intensity reflected the depth of the locking pin into the coil. The pin sensor was calibrated using spacers of known height between the locking pin and the sensing element. All participants used locking pins with a 3.0-mm distance between the raster notches. Example calibration results are shown in Supplementary Fig. S5 online.

In auto and manual modes, a smartphone application operating on an Android phone (Moto 5G, Motorola, Chicago, Illinois) allowed the user to make panel adjustments (Fig. 1e). Users were able to adjust the socket panels inward (TIGHTER button) or outward (LOOSER button) in discrete steps at any time. The same radial adjustment (0.25 mm) was made on all three panels with each button push. If the participant executed an adjustment during walking in auto mode, the controller set point was adjusted by 0.25 mm in the direction of adjustment. The socket size was not communicated to the participant in this study because we expected that knowledge of the socket size would affect their adjustments. We wanted participants to adjust size based on how the socket felt, similar to a traditional socket. Participants could execute a socket release, moving the panels to their largest radial distance, by pushing the RELEASE button in the application. We did not include a RETURN button to move the panels back to their previous position because we were concerned this feature would dominate participant self-reported preferences. In the current study, we sought to focus user feedback to the presence and non-presence of auto adjustment. In auto mode, the smartphone application included an AUTO button to disable automatic adjustments and turn the button gray for 1 h.

### Electronics

The electronics were contained within a small box fastened to the participant’s pylon. An ARM microcontroller continuously sampled the socket sensors and pin sensor via an inter-integrated circuit (I²C interface) (Fig. 1f). Thermistor and motor current data were input through an analog-to-digital converter and then the I²C interface. A Bluetooth connection was used to communicate to and from the smartphone application in auto and manual modes, and a serial peripheral interface was used to transfer sensor and motor position data to a secure digital card. The control system on the microcontroller received position data via the three motor encoder interfaces and communicated commands to the motor driver integrated circuits to adjust panel positions. The sensor and motor data and a log file of the smartphone application commands were stored to the card.

### Controller

The controller automatically adjusted the socket panels to compensate for changes in the SFM. The socket fit control loop relating the socket fit set point to the SFM was based on that from our single-motor adjustable socket^30^. In the present study, the system was reconfigured to operate the 3-motor socket, and enhancements were made to improve reliability. The average of the stance phase minimum for the posterior medial mid-limb and posterior lateral mid-limb sensors was used to calculate the SFM. Uncontrolled changes in the SFM caused by exogenous disturbances, such as changes in limb volume, were to be rejected by the controller (Supplementary Fig. S6 online). Open-loop experiments showed that a first-order linear model of the limb/socket system was adequate for design purposes^30^. A proportional-integral (PI) controller was implemented to adjust socket size based on the SFM.

The controller was active only during walking and operated as illustrated in Supplementary Fig. S6 online. A peak-to-trough amplitude threshold in the anterior distal sensor was used to identify steps. Once 5 sequential steps were detected with no more than 5 s between them, the controller was turned on. The SFM error, the absolute difference between the present SFM and the set point, was calculated and input to an infinite impulse response (IIR) filter with reverse coefficients [*a*_*0*_,* a*_*1*_] = [1, − 1] and forward coefficients [*b*_*0*_,* b*_*1*_] = [*k* * *a*,* k* * (− 2 + *α*)] where *k* was the quotient of 0.6 and the plant gain, and *α* was 1.00167. *α* was derived from the sampling rate (32 Hz) and the controller time constant (*τ* = 10 s). The plant gain, slope of a plot of SFM and socket size (the radial position of the panels in mm), was determined for participants at the outset of their protocol using an open-loop ramp test conducted on a treadmill. The output from the IIR filter, the change to be made in socket size, was communicated to the motors to move the panels, provided it was at least 0.20 mm. If 5 s elapsed without a walking step or if the sensor amplitude reduced below the amplitude threshold for step detection, the controller was turned off. If the controller was turned off, the integral error was carried over to the next walk to avoid a rebound towards the base size at the start of the next bout if the set point was not reset. The set point was reset to the current SFM value at the onset of a walking bout if the SFM error was greater than the product of the plant gain (counts/mm) and 1.00 mm radial panel displacement. The reset was executed to reduce controller instability at the outset of a bout.

Limits were put in place to avoid unsafe conditions. To avoid large rapid changes in panel position, the maximum panel position change while the set point remained unchanged was limited to ± 2.00 mm. Because we found in preliminary testing that panel positions during walking greater than + 2.00 mm from flush caused the panels to lose contact with the liner, we limited panel positions to less than + 2.00 mm during walking. Panel positions greater than + 2.00 mm were however permitted during sitting, allowing execution of socket release.

A hysteresis function was implemented to prevent frequent socket size adjustments when the output from the control algorithm to the motors was near the threshold for execution of a socket size change. This addition prevented the motor from oscillating back and forth between the two sizes, enhancing battery life.

### Study protocol

Participants’ first visit to the research lab was an intake and clinical evaluation session. After informed consent was obtained, the research prosthetist conducted a physical exam to ensure that participants met the inclusion criteria and to record their residual limb and socket physical characteristics. Demographic information and a health history were collected, and participants were fit for their investigational liner. While these procedures were conducted, a researcher scanned the participant’s traditional socket shape. Over the following 2 to 4 weeks, the research team manufactured and calibrated the investigational socket.

The investigational socket was configured in one of three modes: locked, manual, or auto. The motors, frames, and electronics were present in each mode to maintain the weight and size of the investigational prosthesis. In locked mode, the panels were fixed in place flush with the socket wall. In manual mode, the participant was provided a smartphone with the application that allowed the panels to be moved inward and outward as desired (Fig. 1e).

Originally, the order of mode testing was randomized, and we included a 2-week washout period between modes. However, because of IRB regulations introduced by the COVID-19 pandemic partway through the study, the protocol was changed to conduct either the locked or manual mode first and the auto mode last. Take-home use was shortened to approximately 1 week and washouts were not conducted. We began the protocol with locked and manual modes as the investigational prosthesis could be delivered contactless (we drove to participants’ locations, placed the investigational prosthesis on their doorstep, returned to our vehicle, and then communicated by phone with the participants to don the socket and make sure they operated it properly). The auto mode required that the user visit the lab to conduct a 5-min plant gain test on the treadmill, which was only allowed by our IRB under certain conditions. The panels were adjusted in 0.25-mm increments every 8 s across the participants’ acceptable socket size range. The plant gain test established the gain setting (slope of the SFM and socket size plot) which was then programmed into the controller for auto mode.

Participants wore the investigational prosthesis for about a week in each mode. Before starting each mode, participants underwent training and took a quiz to ensure they operated the socket properly. The instructional videos and quizzes were put on the smartphone. Participants were also asked to rate their socket comfort three times a day by text or email, whichever they preferred. Socket comfort was rated with the socket comfort score (SCS), which asks the participant, “On a scale of 0–10, if 0 represents the most uncomfortable socket fit you can imagine, and 10 represents the most comfortable socket fit, how would you score the comfort of your present socket fit”^42^. At the end of testing, participants completed a self-report questionnaire rating their experience for each mode and rank ordering the ease in maintaining fit for the three modes. They were also asked to rank the following variables of the investigational prosthesis from most important to least important: physical size; noise; weight; battery life; visual appearance; ease of use; and smartphone app control. They were also asked open-ended questions about changes they thought should be made to the investigational prosthesis.

### Data analysis

Data from the investigational prosthesis stored to the SD card were uploaded at the end of each test period and segmented into prosthesis days. The start of a prosthesis day was defined as a donned period longer than 30 min. If a donned period shorter than 30 min occurred < 60 min before the first donned period, then it was considered the start of the prosthesis day. The end of the prosthesis day was a doff period longer than 60 min such that there were no donned periods longer than 30 min between this point and the start of the next prosthesis day. Calibration data were used to convert socket sensor, pin sensor, and panel position data to units of distance (mm).

Doffs and steps within the prosthesis day were identified using the data from the socket and pin sensors^57^. A pin sensed distance greater than 15 mm indicated that the pin was no longer engaged in the shuttle lock and the prosthesis was partially or fully doffed. Steps were identified using the walk detection algorithm operating on the anterior distal sensor as described above for the on-board controller except that steps in bouts of less than 5 steps were recorded. For each participant and mode, a median was calculated for each of the following metrics: prosthesis day duration in hours (start-of-day don to end-of-day doff), hours/day conducting walking bouts of 5 or more steps, hours/day doffed, step count/day, and percentage of prosthesis day spent wearing the prosthesis but not conducting walking bouts. Walking bout durations were calculated.

The number of button presses made to adjust the panels was determined from the smartphone application log, and a median and range of button presses/h calculated for the auto and manual modes. The time spent at each pin notch setting was calculated from the pin sensor data, and the percentage of the total prosthesis day time at each pin notch was determined. The time spent within 1.00-mm wide panel distance bins was calculated from the motor encoder data, and the percentage of total prosthesis day time at each panel distance was determined.

The operational effectiveness of the controller was judged by its ability to maintain the SFM set point. Typically, the measured SFM and its set point are plotted as functions of time. To gain insight into the changes in error as a bout progressed, we plotted the average integral absolute error (IAE), estimated as:

$$\overline{IAE} = \frac{1}{N}\mathop \sum \limits_{i = 0}^{N} \left| {SFM_{0} - SFM_{i} } \right|$$

where $SFM_{0}$ is the SFM set point, $SFM_{i}$ is the measured SFM of the *i*th temporal index, and *N* is the number of data points in the walk. We classified the curves into three groups (Fig. 5d): *Convergent* responses were defined as a rise to a maximum followed by a decrease that over time approached an approximately constant value. The constant value was of greater magnitude than at the beginning of the bout. *Divergent* responses were defined as a rise over time that did not maximize. *Assistive* responses were similar to convergent responses except that the constant value they approached, but did not always reach, was of lower instead of higher magnitude than at the beginning of the bout. Convergent and assistive responses are typical of a well-functioning control system, while a divergent response indicates a lack of control.

For the auto mode we calculated the percentage of time during walking bouts that the system was operational, i.e., the system was under active control and the controller rules did not stop panel adjustment. For bouts where panel adjustment was temporarily stopped, we calculated the percentage of those bouts that were stopped because: controller rules at the panel loosen limit were violated; controller rules at the panel tighten limit were violated; or the participant temporarily turned off the controller using the smartphone application. For both the auto and locked modes, we calculated the number of convergent, divergent, and assistive response bouts.

SCS data were processed in accordance with the developer’s instructions^42^. A mean SCS was calculated for each day and the days were plotted over time. For each participant, a median SCS and range were calculated for each mode. Participants’ rankings of socket feature importance were tabulated where a 1 was most important and a 7 was least important. Results from the open-ended interview questions were grouped by themes and summarized in tables.

### Ethical statement

The study was performed under the approval of the Institutional Review Board of the University of Washington (Study #49624).

### Supplementary Information

Supplementary Video 1.

Supplementary Information 1.

Supplementary Information 2.

## Data Availability

All data generated or analysed during this study are included in this published article (and its Supplementary Information files).
