# Supplementary material for: An adaptive prosthetic socket for people with transtibial amputation
Source: Sci Rep. 2024 May 15;14:11168. doi: 10.1038/s41598-024-61234-9 (PMC11096356; doi:10.1038/s41598-024-61234-9)
Supplement: Supplementary file 2 — Supplementary Information 1. [file 41598_2024_61234_MOESM2_ESM.pdf]

“An adaptive prosthetic socket for people with transtibial amputation”

Joan E. Sanders*, Andrew C. Vamos, Joseph C. Mertens, Katheryn J. Allyn, Brian G. Larsen, Daniel Ballesteros, Horace Wang, Nicholas S. DeGrasse, Joseph L. Garbini, Brian J. Hafner, Janna L. Friedly

Supplementary Table S2. Participant characteristics.

**Partic.**	**Reason for Amp.**	**Gender**	**Age**	**Time since Amp.**	**BMI (kg/m^2)**	**RL Length**	**Mid-Limb Circumf.**	**Shape (cylindrical, bulbous, conical)**
P1	trauma	M	59	13	30.1	13.3	27.2	conical
P2	trauma	M	57	30	32.1	11.5	35.1	conical
P3	trauma	M	35	15	36.6	18.0	33.8	conical
P4	trauma	F	39	16	22.7	14.0	24.9	bulbous
P5	trauma	M	45	17	21.2	16.3	28.6	conical
P6	trauma	M	78	42	27.2	18.1	28.8	conical
P7	trauma	M	60	36	23.6	18.5	27.6	conical
P8	trauma	F	64	42	24.1	9.0	22.2	conical short bony
P9	vasc., DM	M	65	4	39.2	19.0	31.3	cylindrical
P10	trauma	M	75	47	26.2	12.3	35	conical
P11	trauma	M	56	4	37.4	16.2	32.3	cylindrical
P12	trauma	M	46	7	24.6	15.6	30.7	cylindrical

Partic.= participant, Amp.= amputation, BMI= body mass index, RL= residual limb, Circumf.= circumference, vasc.= peripheral vascular disease, DM= diabetes mellitus, M= male, F= female

**Partic.**	**Co-Morbidities**	**Socket Volume (PTB to end) mL**	**Plant Gain (counts/mm)**
P1	gout, HBP, high cholesterol	1235	4327
P2	HBP, phantom pain	1331	3774
P3	none	1549	NA
P4	none	1269	3049
P5	none	1456	7370
P6	HBP, brain cancer treatment	1288	NA
P7	none	1333	3464
P8	stroke, HBP, DM, smoker	805	3108
P9	DM, HBP, kidney failure, high cholest.	1550	3105
P10	HBP	907	4185
P11	DM, HBP, smoker	1728	1139
P12	HBP, smoker	1620	2258

Partic.= participant, PTB= patellar tendon bar, HBP= high blood pressure, DM= diabetes mellitus, cholest.= cholesterol

Supplementary Figure S3. Participant supplemental activity data for each mode.

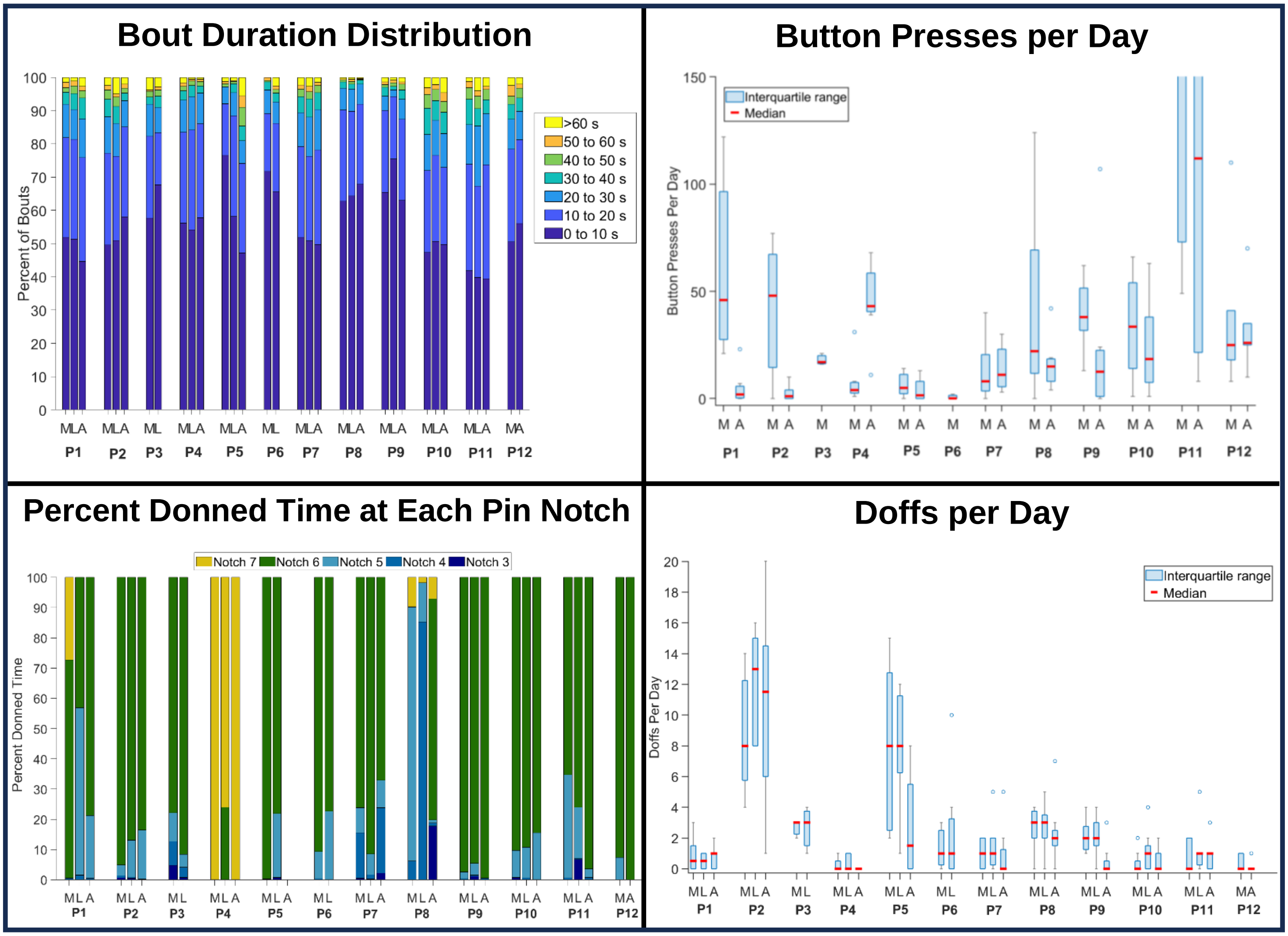

M= manual, L= locked, A= auto, P[#]= participant number

Supplementary Table S4. Participant self-report results.

End of study participant responses

Participant	Would use prosthesis with smartphone application adjustments	Smartphone application adjustments would limit issues with SOCKET FIT	Would like smartphone application to monitor SOCKET FIT	Would like smartphone application to provide details on SOCKET FIT	Would like to share info with others SOCKET FIT	Would prefer remote fob over smartphone
P1	Disagree	Disagree	Neither agree nor disagree	Neither agree nor disagree	Neither agree nor disagree	Disagree
P2	Strongly Agree	Strongly Agree	Strongly Agree	Strongly agree	Neither agree nor disagree	Neither agree nor disagree
P3	Strongly Agree	Agree	Agree	Neither agree nor disagree	Strongly Agree	Neither agree nor disagree
P4	Agree	Neither agree nor disagree	Agree	Agree	Agree	Strongly Agree
P5	Strongly Agree	Strongly Agree	Strongly Agree	Strongly Agree	Strongly Agree	Neither agree nor disagree
P6	Agree	Agree	Neither agree or disagree	Strongly agree	Agree	Neither agree nor disagree
P7	Neither agree nor disagree	Neither agree nor disagree	Disagree	Neither agree nor disagree	Agree	Neither agree nor disagree
P8	Neither agree nor disagree	Agree	Agree	Strongly agree	Agree	Strongly Agree
P9	Agree	Agree	Agree	Agree	Agree	Disagree
P10	Strongly Agree	Agree	Strongly Agree	Strongly agree	Strongly Agree	Strongly Disagree
P11	Strongly Agree	Strongly Agree	Agree	Neither agree nor disagree	Agree	Strongly Agree
P12	Agree	Agree	Agree	Neither agree nor disagree	Agree	Neither agree nor disagree

Themes and responses from open-ended interviews

Partic.	Weight + Size
P1	Less weight
P2	Good start, now miniaturize it. It needs to be a bit lighter
P4	Socket size limited my clothing choices
P5	Weight and Size was least favorite
P10	Knocking into things with panels + brackets - would come down on edge of 5-gallon pail and be "air surfing" and hung up on it<br>Cannot kneel<br>Cannot get pants over it
P11	Not so bulky. If it was lighter it would be so much better
P12	Not being able to wear pants over it, so the physical size. I would use something like this if it was more practical. Size and weight make the difference in use. I would not wear it to work in or be active. Only casual/normal activity
**Partic.**	**Battery Management**
P2	Wireless charging. Did not like charging at night
P3	Battery bar on app for leg % of power
P5	Replaceable batteries or have battery life indicator. Being able to have a second set of batteries charged on me would be good
P8	Battery power made me nervous
**Partic.**	**Changes to Controller**
P2	Remember set positions and go to those positions with one push of button
P3	Individually adjust for panel and release. Neutral 0 setting like release only it tightens to the position for quick donning
P4	If I manually override the auto adjustment, I want it to stay where I put it until my limb changes size for a longer period of time. It's currently too sensitive to the short changes that occur with non-walking movements, like stairs & side stepping. I disliked that auto mode didn't seem to know what fit I wanted
P5	I didn't have to think about it when in auto mode<br>Different modes and smaller + bigger increments. I felt the motors were working too often. If the incremental changes were bigger, the motors wouldn't go on/off as often
P10	Would like the seated auto panel release for relief

Partic.	Interface/Display
P2	Control panel on the limb and on the phone. I like the two-step method to release, it makes it safer to have to push the button twice
P4	I also would want a watch to make quick adjustments instead of having to empty my hands to pull out the phone
P7	Do not like having the phone app with you to make the adjustments (would not want to have an accident and break or loose it!)
P8	Instead of phone I would prefer if something like phone can be attached to prosthetic leg
P11	Remote key pad
**Partic.**	**Controlling Fit**
P2	BEST: The constant good fit
P4	BEST: I like that I could adjust the panels manually
P7	BEST: Simple to control the fit when you want or need to
P8	BEST: Using app to adjust comfort
P9	I liked the automatic because it did most of the work but it still allowed me to fine tune the fit. But I do like the total control of the manual, but I think I still prefer the automatic
P10	BEST: Ease of adjustment not needing to remove pants
P12	BEST: Being able to adjust the panels
**Partic.**	**Other**
P7	The manual release button would be easier to reach, on the inside of the prosthesis
P9	The dependance on the pads --when the pads are adjusted to be tight you really feel the pads. There are 3 points of pressure<br>Something that would make the prosthetic feel like a continuation of the leg - I felt more of the pads as I tightened the socket<br>Want a flexible inner liner for global adjustment

P[#]= participant number

Supplementary Figure S5. Instrumentation.

Socket Sensor

Diagram showing the location of the inductive sensor, antenna, and target in the investigational socket

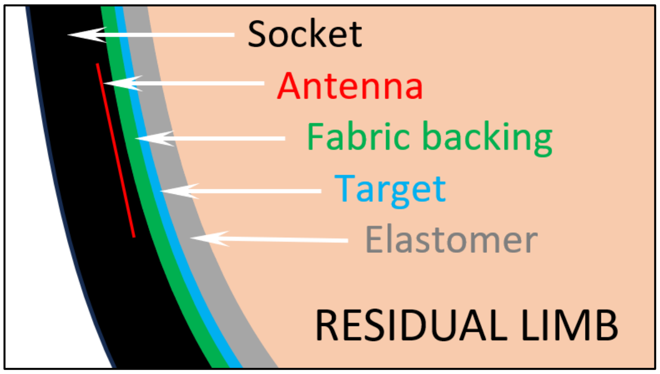

Example socket sensor calibration result

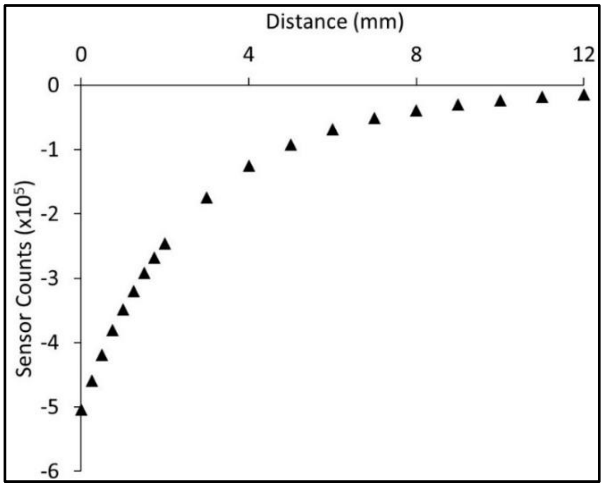

Pin Sensor

Pin sensor with locking pin in the foreground

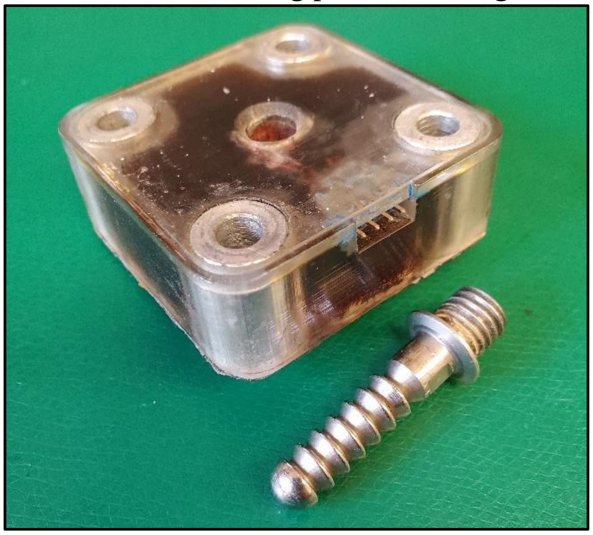

Example pin sensor calibration result

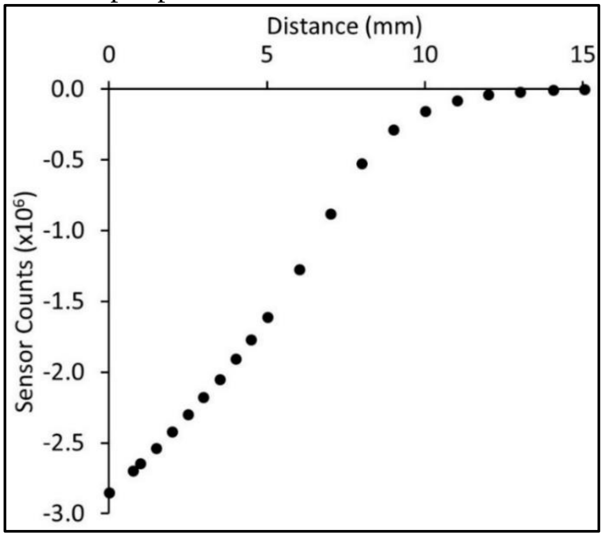

Supplementary Figure S6. Controller design and operation.

The socket fit control loop is shown at the left below. It relates the socket fit set point $SFM_{ref}(s)$ to the measured $SFM(s)$. Uncontrolled changes in the $SFM(s)$ caused by external factors (disturbances $D(s)$), for example limb volume changes, are to be rejected by the controller. The model parameters are the gain (K), which is the effective slope measured during the plant gain test on each participant, and the time constant (τ), which is measured from step responses and is the same for all participants.

As shown at the right below, an increase in limb volume effectively shifts the curve downward, reflecting the tighter fit and decreased SFM. The controller responds by increasing the socket size, traveling up the shifted curve, to restore the SFM.

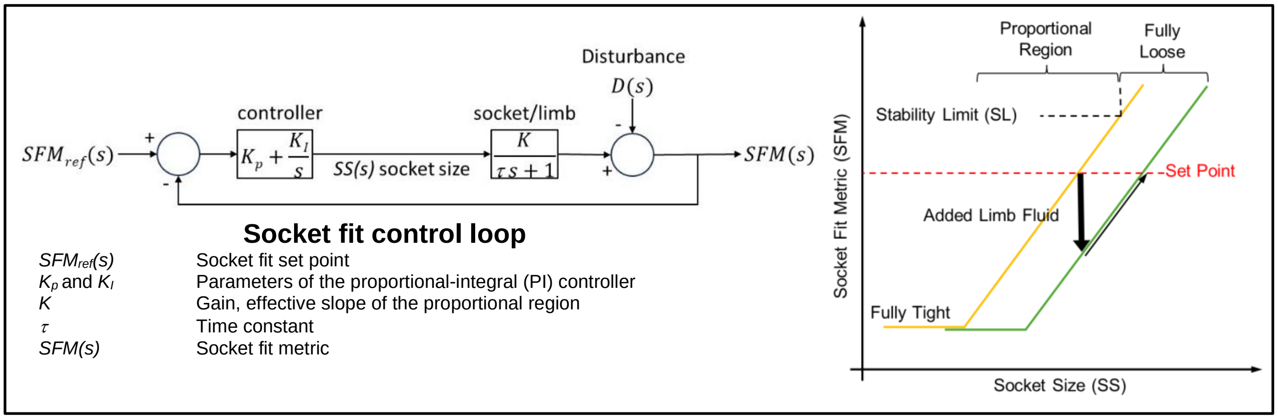

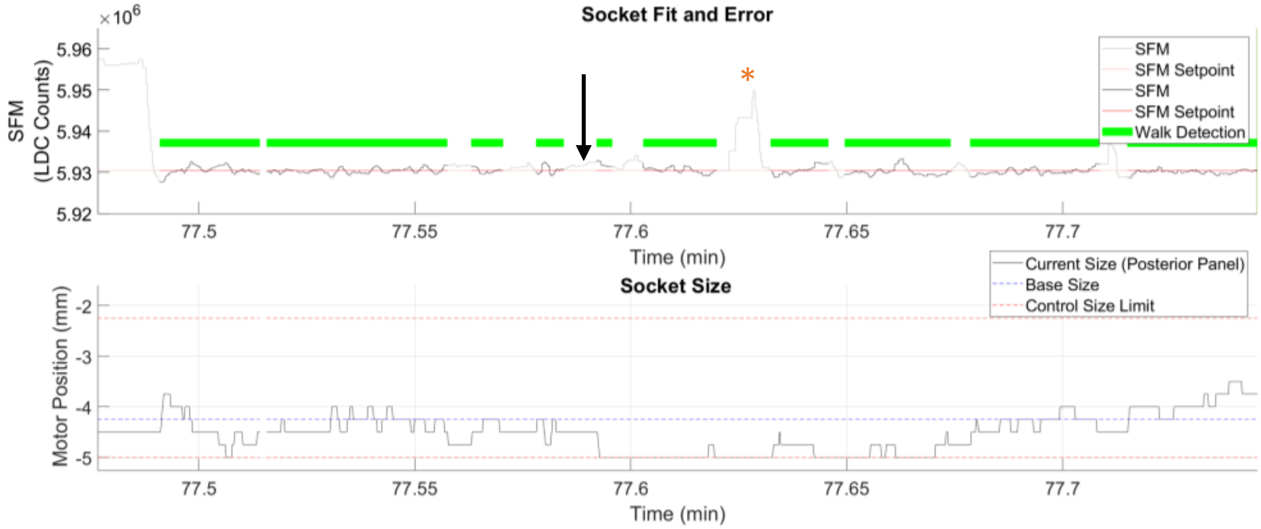

Example controller data. Intermittent walking, standing, and sitting. At the outset the participant is sitting, and then walks intermittently until about 77.63 h where he sits (orange *). The green lines indicate walking. The black arrow indicates a stand.
